# Surface States Induced Photoluminescence Enhancement of Nitrogen-Doped Carbon Dots Via Post-Treatments

**DOI:** 10.1186/s11671-019-3008-9

**Published:** 2019-05-24

**Authors:** Xian Wei, Shiliang Mei, Dan Yang, Guilin Zhang, Fengxian Xie, Wanlu Zhang, Ruiqian Guo

**Affiliations:** 1 Engineering Research Center of Advanced Lighting Technology, Ministry of Education; Institute for Electric Light Sources, Fudan University, Shanghai, 200433 China; 2 Institute of Future Lighting, Academy for Engineering and Technology, Fudan University, Shanghai, 200433 China

**Keywords:** Carbon dots, N-doped, Surface states, PL enhancement, Solvent-dependent effect, Reduced-reaction effect, Metal-enhanced effect

## Abstract

The tunable photoluminescence (PL) of nitrogen-doped carbon dots (NCDs) has attracted much attention in recent years while the specific mechanism is still in dispute. Herein, NCDs with yellow emission were successfully synthesized via a facile hydrothermal approach. Three kinds of post-treatment routes were investigated to verify the influence of surface states on the PL emission of NCDs including solvent-dependent, reduced-reaction and metal-enhanced effect. The interaction mechanism was studied by absorption spectrum, structural characterizations, steady-state and time-resolved spectroscopy. When dispersed in different solvents, the as-prepared NCDs show tunable emission and PL enhancement attributed to hydrogen bonding between solvents and NCDs. Besides, the addition of NaBH_4_ can induce the reduction of the C=O bonds existing in original NCDs to C–O bonds and thus result in the enhancement of the intrinsic (*n*–π*) emission. Moreover, metal-enhanced fluorescence of NCDs can also be observed when adding Ag^+^ into initial NCD solution, which might be ascribed to aggregation-induced emission enhancement. These results for post-treated NCDs demonstrate that surface functional groups are responsible for PL emission and provide new possibilities like multi-image sensing and lighting application.

**Electronic supplementary material:**

The online version of this article (10.1186/s11671-019-3008-9) contains supplementary material, which is available to authorized users.

## Background

In recent years, carbon dots (CDs) have been regarded as a new class of nanoscale light-emitting material with remarkable chemical properties like tunable emission and great biocompatibility [1, 2]. Compared with traditional quantum dots (QDs) such as III–V group QDs (InP) [3, 4], II–VI group QDs (ZnSe) [5, 6], and alloy QDs (ZnInS, CuInS) [7, 8], CDs show environmental friendliness without heavy metal element, facile synthetic routes, and a wide range of raw materials such as citric acid [9], fruit [2, 10, 11], and food [12]. Therefore, the CDs have the potential to be applied in a wide range of fields like bioimaging [2], LED displays [13], fluorescent sensors [10], and photodetector [14–17].

For a long time, the maximum emission of CDs has been limited to the blue region. Though some reports claimed that a longer emission wavelength of CDs was actually realized by different excitation wavelengths, which was not a truly tunable emission. Besides, the intensity of the shifted emission was weaker than the dominant emission, which confined further applications of CDs [18, 19]. Besides, the photoluminescence (PL) origin of CDs is still in dispute, which has been mainly ascribed to intrinsic emission and surface defect emission [9, 20]. To address this issue, it is necessary to develop facile post-treatments to control PL properties and verify the role of surface states through post-treatments in this work.

Up till now, it has been known that surface states could affect chemical, optical, and electronic properties [21–23]. Lan et al. reported that surface passivation could enhance the optoelectronic properties of halide perovskites [22]. Besides, surface acid-base properties also contributed to promoted catalytic capability of CeO_2_ [23]. Recently, solvent-dependent phenomenon of CDs has attracted much interest and the effect brought by solvents to surface states was studied. Chen et al. reported that their NCDs exhibited a tunable emission from a blue to green region in solvents with various polarities [24]. Apart from CDs, carbon nanomaterials like carbon nanosheet have also been reported to exhibit tunable PL in varying solvents, which require several synthetic precursors [25]. Therefore, the solvent-dependent effect is an effective post-treatment to optimize the PL properties of NCDs and further research is required to understand the influence of the interaction between solvents and surface states on the PL properties of NCDs.

Since CDs have been known for numerous surface states, it is possible to develop a facile approach for post-treating NCDs with some reduction reagents and metal cations to modify the PL properties. For example, Hu et al. has reported that NaBH_4_ could be used during carbonization to realize tunable peak emissions of CDs [26]. However, the synthetic process which involved several procedures to form gel was quite complex. Apart from surface reduction, metal-enhanced effect brought by aggregation-induced emission enhancement (AIEE) could occur when metal ions are added into fluorescent materials [27, 28]. Wang et al. have synthesized CDs modified by glutathione. The variation of surface charge between CDs caused by Fe^3+^ could lead to different degrees of aggregation and PL enhancement [28]. Further characterizations are still required for the reasonable mechanism of the PL properties of NCDs treated by metal ions.

In this work, NCDs with yellow emission were successfully synthesized via a facile hydrothermal approach. Three kinds of post-treatment routes via solvent-dependent, reduced-reaction and metal-enhanced effect have been utilized to investigate the relationship between PL properties and surface states of NCDs. The as-prepared NCDs in different solvents show tunable emission and PL enhancement attributed to hydrogen bonding between solvents and NCDs. Besides, the addition of NaBH_4_ can induce the reduction of the C=O bonds existing in original NCDs to C–O bonds and thus result in the enhancement of the intrinsic (*n*–π*) emission. Moreover, metal-enhanced fluorescence of NCDs can also be observed when adding Ag^+^ into initial NCD solution, which might be ascribed to aggregation-induced emission enhancement. Through three kinds of post-treatments, the role played by surface states in PL emission is discussed, and the results help to better understand the chemical nature of the observed NCDs.

## Methods

### Chemicals

O-Phenylenediamine (OPD, 99.9%), all the organic solvents of water, ethylene glycol (EG), ethanol, dimethyl sulfoxide (DMSO), acetone, and toluene were of analytical grade and purchased from various commercial companies. Metal chlorides/nitrates and 4-(2-hydroxyerhyl)piperazine-1-erhanesulfonic acid (HEPES) were purchased from Aladdin. They were all used directly and without further purification. Water was deionized and purified by being passed through a Milli-Q water purification system.

### Synthesis of NCDs

Concretely, 0.05 g OPD was dissolved in 10 mL of Milli-Q water under vigorous stirring until the solution became clear. Then, the mixture was transferred into a 25-mL Teflon-lined stainless steel autoclave and heated at 180 °C for 6 h in an electric oven. After drastic reaction, the obtained claybank solution was centrifuged at 9000 rpm to remove the precipitate and the supernatant was freeze dried for 24 h in a vacuum freeze dryer to gather the light yellow powder.

### Post-Treatment

0.01 g of the as-prepared NCD powder was dispersed into 10 mL of water, EG, ethanol, DMSO, acetone, and toluene respectively in order to investigate the solvent-dependent effect. In addition, different concentration of NaBH_4_ solution (10 mL) ranging from 0 to 0.04 g/mL was prepared and reacted with NCD powder to collect PL and ultraviolet-visible (UV-vis) absorption spectra. The fluorescence titration measurements were carried out by adding NCDs and 50 μM of different metal ions including Ag^+^, Cd^2+^, Cs^+^, Cu^2+^, Fe^3+^, In^3+^, Mg^2+^, Mn^2+^, Pb^2+^, and Zn^2+^ into pH 7.2 HEPES-buffered water (10 mL). Furthermore, NCDs were treated with different concentrations of Ag^+^ from 0 to 300 μM.

### Characterizations

The obtained NCDs were characterized by high-resolution transmission electron microscopy (HRTEM; JEM-2s100F, JEOL, Japan), X-ray diffraction (XRD; D8 Advance, Bruker, Germany), X-ray photoelectron spectroscopy (XPS; ESCALAB 250XI, Thermo, USA), and Fourier transform-infrared spectroscopy (FT-IR; Nicolet 6700, Thermo Fisher, USA). UV-vis absorption spectra and PL spectra of QDs were recorded using a UV-vis spectrophotometer (759S, Shanghai Lengguang, China) and fluorescence spectrophotometer (F97XP, Shanghai Lengguang, China), respectively. The fluorescence lifetimes of the as-prepared NCDs were measured on a time-resolved spectrofluorometer (FLS 920, Edinburgh Instruments, UK). Zeta potentials were measured by a Zetasizer (Malvern, UK).

## Results and Discussion

### Structure Characterizations

**Fig. 1:**
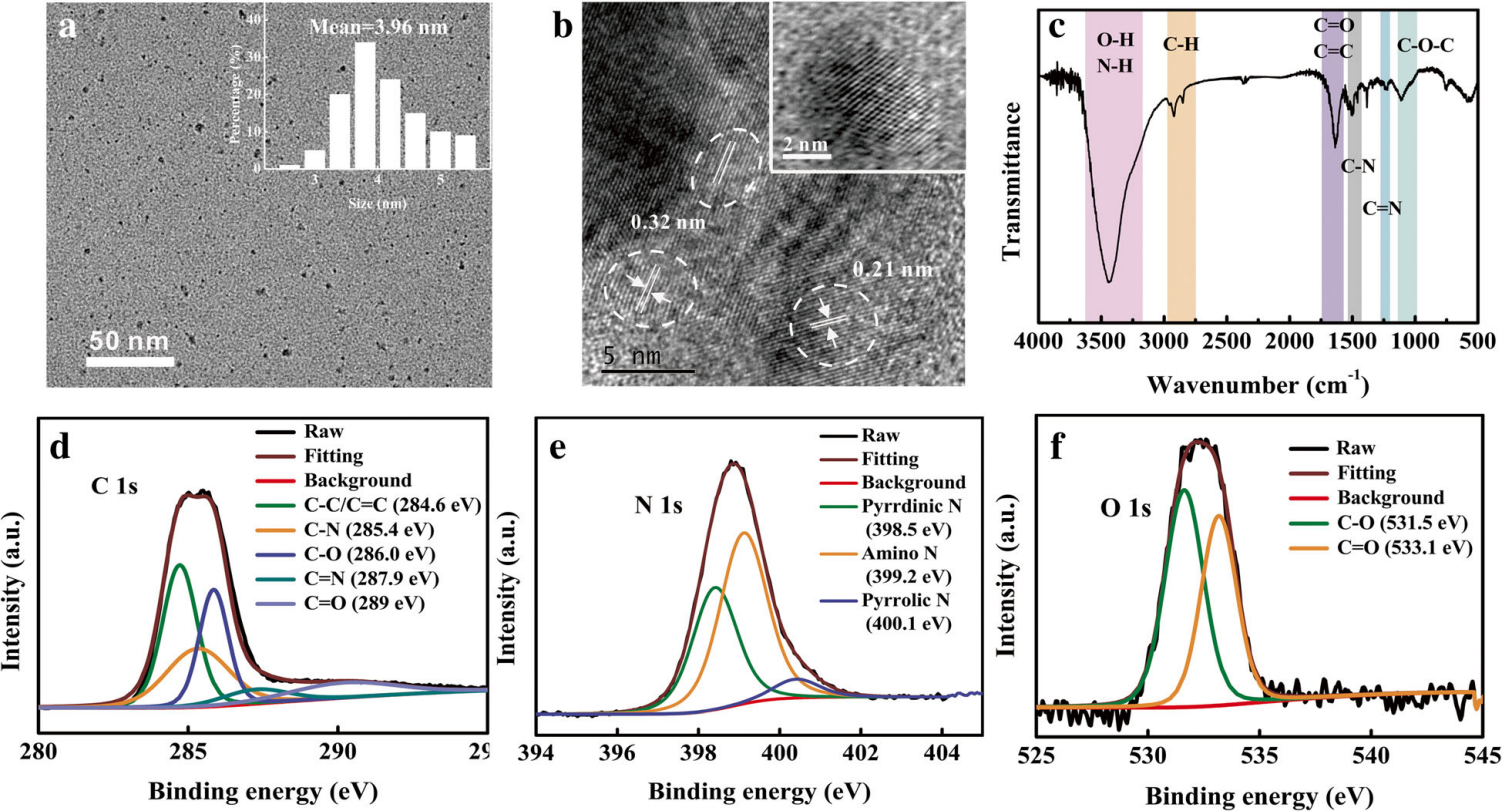
**a** TEM and **b** HRTEM images and **c** FT-IR spectra of NCDs. High-resolution **d** C 1s, **e** N 1s, and **f** O 1s spectra of NCDs. The histogram of size distributions and HRTEM image of a single QD are shown in the inset at high magnification while the scale bar is 2 nm

The morphology and size of NCDs in water are studied by TEM as shown in Fig. 1. Based on the histogram of size distributions, it can be seen that the size of the as-prepared NCDs is in the range of 3–6 nm with an average diameter of 3.96 nm. In Fig. 1b, it is apparent that the NCDs exhibit crystalline lattice fringes of 0.21 nm and 0.32 nm corresponding to the lattice planes (100) and (002) of graphic carbon [7, 29]. The XRD pattern (Additional file 1: Figure S1) shows a broad peak centered at around 20**°**. This indicates that the NCDs consist of small crystalline cores with a disordered surface [30].

The surface functional groups are investigated by FT-IR as shown in Fig. 1c. The broad absorption band within 3100–3600 cm^− 1^ derives from the stretching vibration of hydroxyl bonds (O–H) and N–H, which could be largely ascribed to the amino group from the OPD [31]. The relatively weaker band located around 1570–1750 cm^− 1^ is assigned to carbonyl bonds (C=O) and aromatic C=C. Besides, the absorption peak centered at ~ 1411 and ~ 1239 cm^− 1^ corresponds to the stretching mode of C–N and C=N, respectively [32, 33]. The existence of C–O–C bonds leads to the absorption band from 990 to 1170 cm^− 1^ [34]. Based on these results, it can be concluded that a lot of functional groups exist on the surface of NCDs, which could further be verified by XPS to investigate the chemical bond compositions. As presented in Additional file 1: Figure S1b, the full-scan XPS spectra have three typical peaks: C 1s (285 eV), N 1s (399 eV), and O 1s (533 eV), which confirms that the as-prepared NCDs consist of C, N, and O elements. Furthermore, the atomic percentages of C, N, and O were 73.81%, 22.59%, and 3.6%, respectively. According to the high-resolution spectra of C 1s in Fig. 1d, there are three peaks corresponding to different states of carbon: the dominant graphitic sp^2^ C–C/C=C (284.6 eV), C–N (285.4 eV), C–O (286.0 eV), C=N (287.9 eV), and C=O (289 eV) [34, 35]. Remarkably, the spectra of N 1s in Fig. 1e indicate the as-prepared NCDs contain rich nitrogen heterocyclic structures, representing pyrridinic N (398.5 eV), amino N (399.2 eV), and pyrrolic N (400.1 eV). The high-resolution spectrum of O 1s is divided into two peaks, which could be attributed to C–O (531.5 eV) and C=O (533.1 eV), respectively [35, 36].

### Solvent-Dependent Effect

Recently, solvatochromism which was originally used in organic dyes has attracted much attention and is rarely studied in NCDs [37]. The interaction between NCDs and solvents is still to be investigated, which may play an important role in understanding the luminescent mechanism of NCDs. It is apparent that the emission peaks of NCDs in different solvents including water, EG, ethanol, DMSO, acetone, and toluene (Additional file 1: Figure S2) exhibit independence of excitation wavelengths, which is unique to traditional reports. This phenomenon may be ascribed to the precursors with high content of N atoms. Based on previous reports, those amino-rich (−NH_2_) CDs might show less excitation anisotropy through interactions between surface functional groups on the distorted sp^2^ carbon framework [9, 38].

**Fig. 2:**
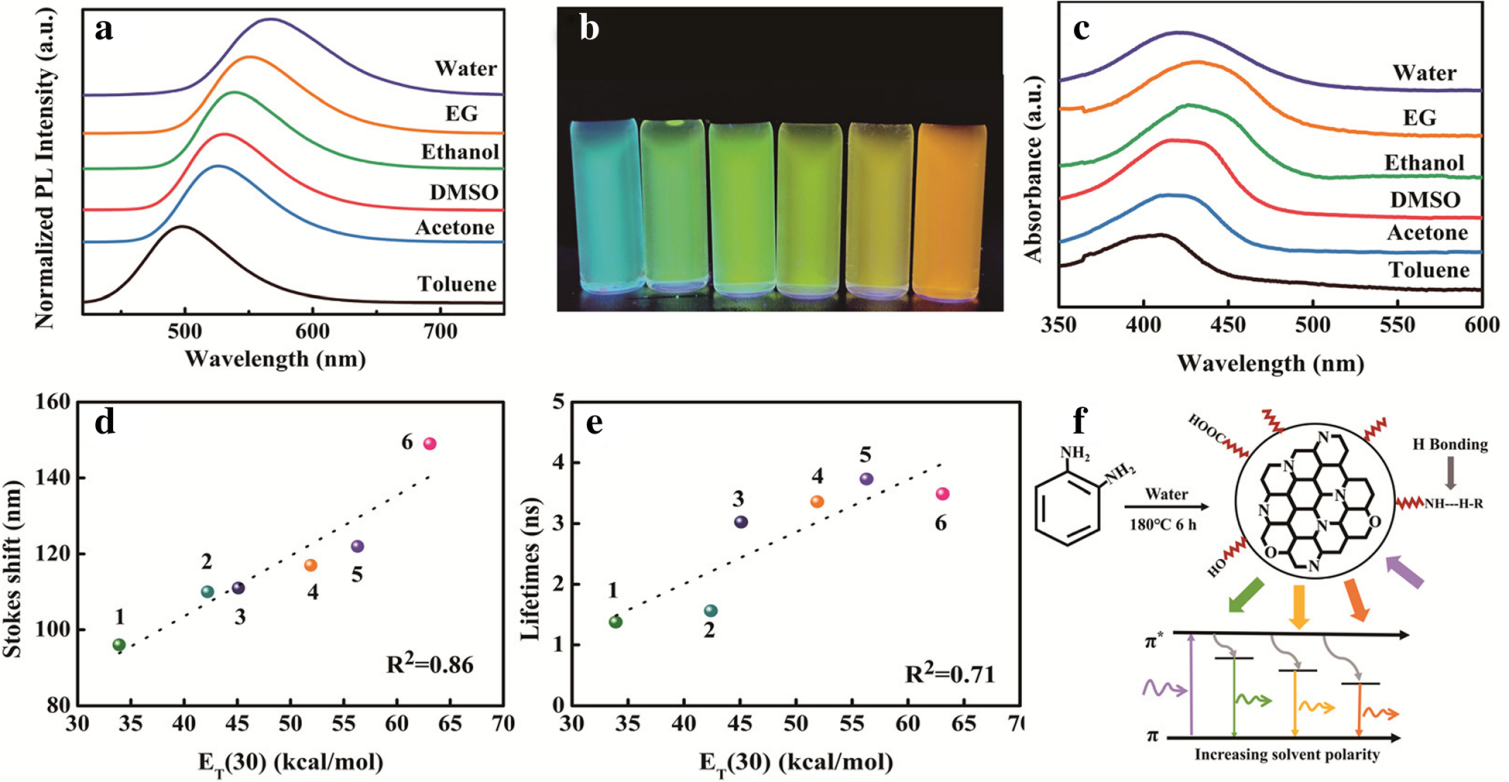
**a** Normalized PL spectra of NCDs in six solvents, *λ*_ex_ = 400 nm. **b** Digital photograph of NCDs in six solvents under an UV lamp. **c** Absorption spectra of NCDs in six solvents. Relationship between the spectral parameters and E_T_ (30): **d** Stokes shifts and **e** lifetimes. **f** Schematic illustration of the interaction between surface functional groups and solvents. Solvents used are as follows. 1: toluene, 2: acetone, 3: DMSO, 4: ethanol, 5: EG, 6: water

In order to investigate the influence brought by solvent-dependent effect to NCDs, the normalized PL spectra in various solvents under the same excitation wavelength are shown in Fig. 2a. The PL emission of NCDs exhibits a red shift when the solvents change from toluene to water. Thus, tunable emission with the peak positions from 500 to 569 nm is procured under a UV lamp (365 nm) as shown in Fig. 2b. The PL intensity of NCDs dispersed in other five solvents gets promoted to different degrees compared with that of NCDs in water (Additional file 1: Figure S3). Besides, the UV-vis absorption spectra of NCDs were also measured in Fig. 2c. It can be concluded that the absorption spectra of NCDs shift towards a long wavelength with the incremental solvent polarity except water, which could be attributed to the low solubility in water [32]. In order to obtain more information about solvent-related properties of NCDs, the PL lifetimes and Stokes shifts of NCDs in different solvents were calculated (Additional file 1: Table S1). The PL decay curves of NCDs can be well fitted by monoexponential function (Additional file 1: Figure S4), indicating the single electronic state of PL emission. Since the absorption band also changes with different solvents, there might be different kinds of electronically excited states, which incur tunable PL emission [26].

Here, we adopted the E_T_ (30) index which was used as classical polarity parameter to study hydrogen bonding (HB) [39]. Functional groups like −OH and −NH_2_ could work as the donor or acceptor of HB. In Fig. 2d and e, the correlation relationships between Stokes shifts, lifetimes, and E_T_ (30) index exhibit certain linear relationships, and specific values are given in Additional file 1: Table S1. It is apparent that the Stokes shifts and lifetimes increase almost linearly with the increasing value of E_T_ (30), which characterizes the ability to form HB [36]. Besides, it has been reported that stronger HB could lead to more interaction between functional groups and different solvents [25, 35]. Specific mechanism and reaction depiction of NCD formation are illustrated in Fig. 2f. According to XPS data, nitrogen-containing sub-structures mainly exist as pyridinic and pyrrolic forms, which indicates the molecular structures inside a sp^2^-hybridized core. Due to numerous functional groups acting as additional electron acceptor and donor, the increased surface electron density provides possibilities for new energy transfer ways [35]. Based on the results in Fig. 2c, it has been concluded that the as-prepared NCDs show the weakest luminescence in water on account of the highest polarity in six solvents. Besides, the emission peaks of NCDs tend to red shift with the increase of solvent polarity which is commonly identified as a solvent-dependent effect. When the solvent polarity increases, the HB between NCDs and six solvents becomes stronger and more surface groups surrounding the carbon nitride crystalline core are involved in the emissive mechanism, therefore resulting in spectral shift of NCDs towards longer wavelengths [36]. Hence, it can be concluded that the tunable emission of NCDs could be obtained through the interaction between NCDs and solvents.

### Reduced-Reaction Effect

**Fig. 3:**
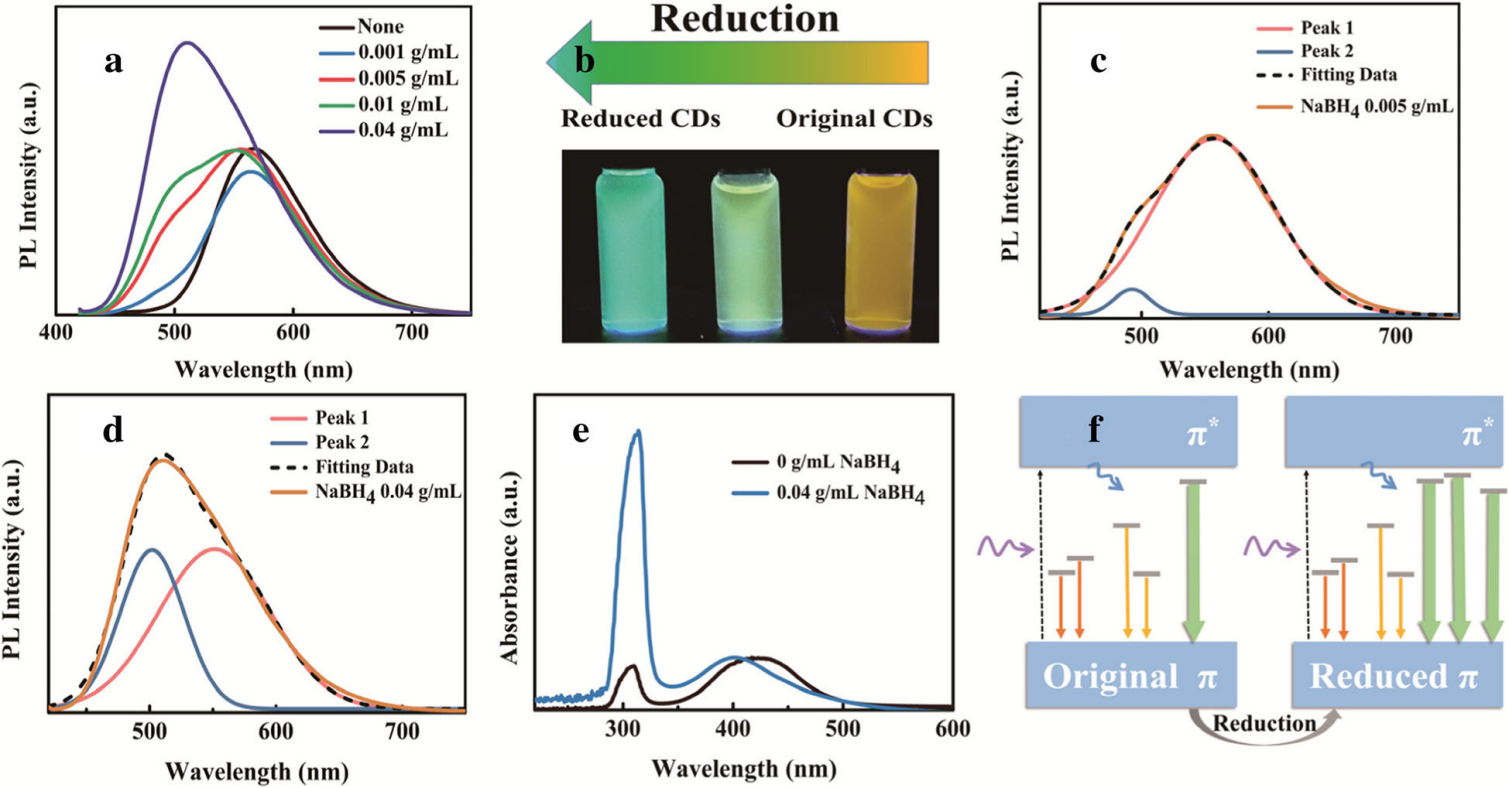
**a** PL spectra of NCDs treated with different concentration of NaBH_4_ ranging from 0 to 0.04 g/mL, *λ*_ex_ = 400 nm. **b** Digital photographs of NCDs treated with 0, 0.01, and 0.04 g/mL NaBH_4_ under an UV lamp. Deconvoluted PL spectra of NCDs treated with **c** 0.005 and **d** 0.04 g/mL NaBH_4_ separately with two emission bands of peak 1 and peak 2. **e** UV-vis absorption spectra of NCDs treated with 0 and 0.04 g/mL NaBH_4_. **f** The schematic illustration of the proposed emission process in the original and reduced NCDs

Since NCDs have numerous surface defect states, it is plausible to modify the functional groups through chemical reaction. So as to further testify the influence of post-treatment on NCDs apart from a solvent-dependent effect, NCDs were treated with different concentrations of NaBH_4_ as a reduction agent ranging from 0 to 0.04 g/mL. The PL spectra are shown in Fig. 3a, and it can be seen that the fluorescence intensity is greatly promoted. Besides, the emission band presents a blue shift from 567 to 510 nm with the increasing concentration of NaBH_4_ (Additional file 1: Figure S5a). The digital photographs of NCDs treated with 0, 0.01, and 0.04 g/mL NaBH_4_ are shown in Fig. 3b to present their PL color. The spectra of NCDs treated with 0.005 and 0.04 g/mL NaBH_4_ are both deconvoluted into two Gaussian-like peaks respectively identified as peak 1 and peak 2 [40]. As shown in Fig. 3c, when raw NCDs are treated with 0.005 g/mL NaBH_4_, the PL spectrum is mainly dominated by peak 1 at around 565 nm with a small peak at 496 nm. The PL spectrum of NCDs with 0.04 g/mL NaBH_4_ in Fig. 3d can be split into two peaks with almost equal intensity. It can be concluded that further adding NaBH_4_ leads to an obvious increase of peak 2 emission intensity while the intensity of peak 1 remains almost unchanged. The specific peak positions and PL intensities of peak 1 and peak 2 under different concentrations of NaBH_4_ are presented in Additional file 1: Figure S5. It is apparent that the intensity of peak 1 maintains at a stable level while that of peak 2 shows a drastic increase (Additional file 1: Figure S5b). In addition, peak 1 tends to move towards shorter wavelengths while peak 2 remains almost unchanged (Additional file 1: Figure S5c).

The element compositions of the reduced NCDs (0.04 g/mL NaBH_4_) were further characterized by XPS (Additional file 1: Figure S6). Compared with raw NCDs, the increasing concentration of NaBH_4_ leads to an incremental proportion of oxygen and less content of nitrogen (Additional file 1: Table S2). The C=O bonds existing in original NCDs might be reduced to C–O bonds after the addition of NaBH_4_. So as to further study the optical properties, the UV-vis absorption spectra of NCDs treated by 0 and 0.04 g/mL NaBH_4_ are shown in Fig. 3e, which present two absorption bands at around 320 and 410 nm. It seems that a higher concentration of NaBH_4_ will lead to a higher intensity of the absorption band centered at approximately 320 nm which resulted from *n*–π* transition, which might be attributed to a higher content of C–O–C and less nitrogen-containing groups [41, 42]. The decreased PL lifetime from 3.48 to 2.2 ns of NCDs after treated by NaBH_4_ (Additional file 1: Figure S7) verified the intrinsic emission derived from (C–O–C) within *n*–π* transition corresponding to the previous work [26].

The feasible energy transfer process about PL of NCDs with incremental concentration of NaBH_4_ is illustrated in Fig. 3f. The as-prepared NCDs without NaBH_4_ treatment may have a large number of different electronic states owing to the oxygen and nitrogen containing groups. Once reduced, the increasing concentration of electronically excited states related to (C–O–C) plays a dominant role in the PL of NCDs based on the XPS results [40]. The electron-hole recombination process related with the increased energy states leads to emission at shorter wavelengths as well as an obvious PL enhancement. Combined with experimental results, the presence of numerous surface functional groups provides possibilities for tunable emission through adjusting the relative proportion of different energy states by means of reduction [43–46].

### Metal-Enhanced Effect

**Fig. 4:**
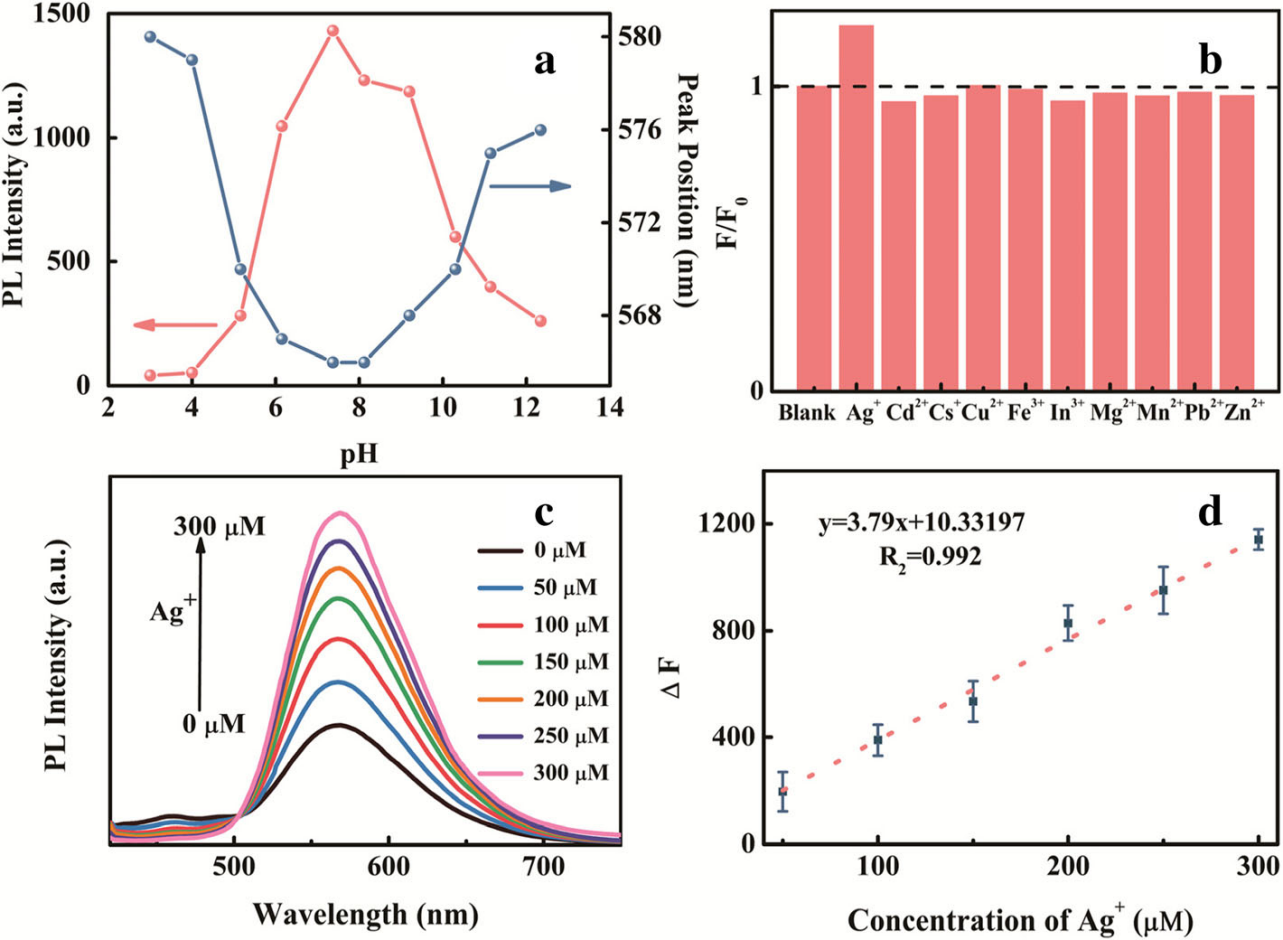
**a** PLs intensity and peak position of NCDs in different pH values. **b** PL intensity ratios (*F*/*F*_0_) of NCDs, *F*_0_ is the PL intensity of the original NCDs, while *F* is that of NCDs treated with different metal ions (50 μM). **c** PL spectra of NCDs treated with the increasing titrations of Ag^+^ from 0 to 300 μM, *λ*_ex_ = 400 nm. **d** Plot of fluorescent intensity of NCDs against Ag^+^ concentration in the range of 0 to 300 μM

Apart from a reduced-reaction effect, introducing metal ions is another effective post-treatment to investigate the PL characteristics of NCDs [47]. In order to exclude the influence brought by pH, the stability of the as-prepared NCDs is detected under different pH values as shown in Fig. 4a. Apparently, the neutral solution is the optimal environment for NCDs, and thus, the HEPES-buffered water solution (pH 7.2) is adopted. We investigate the influence of different metal ions on NCDs by using a fluorescence titration method, and the intensity contrasts are shown in Fig. 4b. Interestingly, Ag^+^ shows a significant effect on PL enhancement among metal ions studied.

More information about the PL spectra of NCDs treated with different concentrations of Ag^+^ is presented in Fig. 4c. The fluorescence intensity of NCDs at 566 nm gradually increases with an incremental concentration of Ag^+^, but the peak position does not change. In an attempt to further study a specific relationship, the plot of fluorescent intensity of NCDs against Ag^+^ concentration is shown in Fig. 4d. It can be concluded that the ∆*F* (*F*–*F*_0_) versus [Ag^+^] exhibits a good linear range from 0 to 300 μM and the coefficient of determination (*R*^2^) is 0.992 [47]. In order to obtain better understanding about this PL enhancement, the PL decay curves of NCDs in the absence and presence of Ag^+^ with the concentration of 200 μM are supplemented in Additional file 1: Figure S8. Subtle change of lifetimes could be found after incorporating Ag^+^ into NCD aqueous solution, which may be ascribed to the formation of a stable complex [48]. According to a previous report, Namasivayam et al. reported that Zn^2+^ could enhance the intensity of CDs due to an association between Zn^2+^ and amine groups (−NH_2_) present on the surface [49]. So as to investigate the surface charge state, the Zeta potential of NCDs in aqueous solution is measured to be − 34.0 mV, which indicates that the surface of NCDs is negatively charged and the NCDs are rather stable. After introducing Ag^+^, the Zeta potential of NCDs changes to − 27.8 mV, of which the absolute value was lower than that in raw solution. Owing to the decrease of mutual repulsion, it is speculated that the PL enhancement might be triggered by AIEE properties for the abundant amino groups based on FT-IR and XPS results [50]. The aggregation of NCDs treated by Ag^+^ is shown in Additional file 1: Figure S9. It seems that the decreasing electrostatic repulsion between the QDs leads to the aggregation, which passivates the surface defect states of NCDs and the PL intensity of NCDs is enhanced [27, 28, 51]. These results provide potential for future application in vivo biological fields and help to further understand the role of surface states to PL properties of NCDs.

## Conclusion

To sum up, NCDs with yellow emission were successfully synthesized via a facile hydrothermal approach and three post-treatments based on solvent-dependent, reduced reaction and metal-enhanced effect have been applied to modify the PL characteristics of NCDs. When dispersed in different solvents, the as-prepared NCDs show tunable emission and PL enhancement attributed to hydrogen bonding between solvents and NCDs. Besides, the addition of NaBH_4_ can induce the reduction of the C=O bonds existing in original NCDs to C–O bonds and thus result in the enhancement of the intrinsic (*n*–π*) emission. Moreover, metal-enhanced fluorescence of NCDs can also be observed when adding Ag^+^ into initial NCD solution, which might be ascribed to aggregation-induced emission enhancement. These results reveal that the interaction between external factors and surface functional groups plays a critical role in PL characteristics, which help to understand the specific PL mechanism.

## Additional file

Additional file 1: **Figure S1.** (a) XRD pattern and (b) XPS survey of NCDs. **Figure S2.** Photoluminescence spectra of NCDs in 6 different solvents of (a) water, (b) ethylene glycol (EG), (c) ethanol, (d) dimethyl sulfoxide (DMSO), (e) acetone, and (f) toluene excited by different wavelengths. **Figure S3.** Relative emission intensity of NCDs. **Figure S4.** Fluorescent decay curves of NCDs in different solvents. **Table S1.** Six solvents used in this study. E_T_ (30) polarity parameter of the solvents. Emission peak, absorption band, Stokes shifts, and lifetimes for the CDs dispersed in each kind of solvents. **Figure S5.** (a) Normalized PL spectra of NCDs treated with different concentrations of NaBH_4_ ranging from 0 to 0.04 g/mL, *λ*_ex_ = 400 nm. (b) PL intensities and (c) peak positions of two peaks deconvoluted from emission spectra of NCDs treated with different concentrations of NaBH_4_. **Figure S6.** (a) XPS survey spectrum of NCDs treated by 0.04 g/mL NaBH_4_. High-resolution (b) C 1s, (c) N 1s, and (d) O 1s spectra. **Table S2.** XPS results of NCDs in the absence and presence of 0.04 g/mL NaBH_4_. The measured atomic ratios of C, N, and O were calculated. **Figure S7.** Fluorescence decay curves of CDs in the absence and presence of 0.04 g/mL NaBH_4_. **Figure S8.** Fluorescence decay curves of NCDs in the absence and presence of Ag^+^ with the concentration of 200 μM (in the HEPES-buffered water solution). **Figure S9.** TEM image of NCDs treated by Ag^+^ (200 μM). (DOCX 32773 kb)

## Abbreviations

AIEE: Aggregation-induced emission enhancement
CDs: Carbon dots
DMSO: Dimethyl sulfoxide
EG: Ethylene glycol
FT-IR: Fourier transform-infrared spectroscopy
HB: Hydrogen bonding
HEPES: 4-(2-Hydroxyerhyl)piperazine-1-erhanesulfonic acid
HRTEM: High-resolution transmission electron microscopy
NCDs: Nitrogen-doped carbon dots
OPD: O-Phenylenediamine
PL: Photoluminescence
QDs: Quantum dots
rpm: Revolution per minute
UV-vis: Ultraviolet-visible
XPS: X-ray photoelectron spectroscopy
XRD: X-ray diffractometer

## References

[1] Ma L, Xiang W, Gao H (2016). Facile synthesis of tunable fluorescent carbon dots and their third-order nonlinear optical properties. Dyes Pigments.

[2] Ding H, Ji Y, Wei JS (2017). Facile synthesis of red-emitting carbon dots from pulp-free lemon juice for bioimaging. J Mater Chem B.

[3] Yang W, He G, Mei S (2017). Controllable synthesis of dual emissive Ag:InP/ZnS quantum dots with high fluorescence quantum yield. Appl Surf Sci.

[4] Zhang G, Mei S, Wei X (2018). Dual-emissive and color-tunable Mn-doped InP/ZnS quantum dots via a growth-doping method. Nanoscale Res Lett.

[5] Zhang J, Chen Q, Zhang W (2015). Microwave-assisted aqueous synthesis of transition metal ions doped ZnSe/ZnS core/shell quantum dots with tunable white-light emission. Appl Surf Sci.

[6] Chen Q-H, Zhang J, Chen G-P, Guo R-Q (2013). Optical control of the spindle-liked ZnSe quantum dots with precursor solvent and Mn doping. Appl Surf Sci.

[7] Zhu J, Mei S, Yang W (2017). Tunable emission of Cu (Mn)-doped ZnInS quantum dots via dopant interaction. J Colloid Interface Sci.

[8] Mei S, Zhu J, Yang W (2017). Tunable emission and morphology control of the Cu-In-S/ZnS quantum dots with dual stabilizer via microwave-assisted aqueous synthesis. J Alloys Compd.

[9] Qu S, Liu X, Guo X (2014). Amplified spontaneous green emission and lasing emission from carbon nanoparticles. Adv Funct Mater.

[10] Wang N, Wang Y, Guo T (2016). Green preparation of carbon dots with papaya as carbon source for effective fluorescent sensing of Iron (III) and Escherichia coli. Biosens Bioelectron.

[11] De B, Karak N (2013). A green and facile approach for the synthesis of water soluble fluorescent carbon dots from banana juice. RSC Adv.

[12] Zhao Y, Zhang Y, Liu X (2017). Novel carbon quantum dots from egg yolk oil and their haemostatic effects. Sci Rep.

[13] Yu WW, Wang Y, Cui T (2013). Color-switchable electroluminescence of carbon dot light-emitting diodes. ACS Nano.

[14] Hu W, Ho JC, Fang H (2016). High-performance ferroelectric polymer side-gated CdS nanowire ultraviolet photodetectors. Adv Funct Mater.

[15] Chen YZ, You YT, Chen PJ (2018). Environmentally and mechanically stable selenium 1D/2D hybrid structures for broad-range photoresponse from ultraviolet to infrared wavelengths. ACS Appl Mater Interfaces.

[16] Lan C, Zhou Z, Zhou Z (2018). Wafer-scale synthesis of monolayer WS_2_ for high-performance flexible photodetectors by enhanced chemical vapor deposition. Nano Res.

[17] Long M, Wu X, Fang H (2018). High-performance near-infrared photodetectors based on p-type SnX (X = S, Se) nanowires grown via chemical vapor deposition. ACS Nano.

[18] Sun YP, Zhou B, Lin Y (2006). Quantum-sized carbon dots for bright and colorful photoluminescence. J Am Chem Soc.

[19] Liu R, Wu D, Liu S (2009). An aqueous route to multicolor photoluminescent carbon dots using silica spheres as carriers. Angew Chem Int Ed.

[20] Li B, Zhu S, Wei H (2011). Strongly green-photoluminescent graphene quantum dots for bioimaging applications. Chem Commun.

[21] Duran Retamal JR, Ho CH, Tsai KT (2019). Self-organized Al nanotip electrodes for achieving ultralow-power and error-free memory. IEEE Trans Electron Devices.

[22] Lan C, Zhou Z, Wei R, Ho JC (2019). Two-dimensional perovskite materials: from synthesis to energy-related applications. Mater Today Energy.

[23] Ma Y, Gao W, Zhang Z (2018). Regulating the surface of nanoceria and its applications in heterogeneous catalysis. Surf Sci Rep.

[24] Chen Y, Zheng M, Xiao Y (2016). A self-quenching-resistant carbon-dot powder with tunable solid-state fluorescence and construction of dual-fluorescence morphologies for white light-emission. Adv Mater.

[25] Choi Y, Kim S, Choi Y (2017). Morphology tunable hybrid carbon nanosheets with solvatochromism. Adv Mater.

[26] Hu S, Trinchi A, Atkin P, Cole I (2015). Tunable photoluminescence across the entire visible spectrum from carbon dots excited by white light. Angew Chem Int Ed.

[27] Yang XX, Wu WB, Li YF (2013). A surfactant-assisted redox hydrothermal route to prepare highly photoluminescent carbon quantum dots with aggregation-induced emission enhancement properties. Chem Commun.

[28] Wang C, Jiang K, Xu Z (2016). Glutathione modified carbon-dots: from aggregation-induced emission enhancement properties to a “turn-on” sensing of temperature/Fe^3+^ ions in cells. Inorg Chem Front.

[29] Nie H, Li M, Li Q (2014). Carbon dots with continuously tunable full-color emission and their application in ratiometric pH sensing. Chem Mater.

[30] Han C, Wang R, Wang K (2016). Highly fluorescent carbon dots as selective and sensitive “on-off-on” probes for iron (III) ion and apoferritin detection and imaging in living cells. Biosens Bioelectron.

[31] Qu S, Wang X, Lu Q (2012). A biocompatible fluorescent ink based on water-soluble luminescent carbon nanodots. Angew Chem Int Ed.

[32] Chao D, Lyu W, Liu Y (2018). Solvent-dependent carbon dots and their applications in the detection of water in organic solvents. J Mater Chem C.

[33] Lin S, He M, Zhang Y (2017). Solvatochromism of bright carbon dots with tunable long-wavelength emission from green to red and their application as solid-state materials for warm WLEDs. RSC Adv.

[34] Mei S, Wei X, Hu Z (2019). Amphipathic carbon dots with solvent-dependent optical properties and sensing application. Opt Mater.

[35] Wang H, Sun C, Chen X (2017). Excitation wavelength independent visible color emission of carbon dots. Nanoscale.

[36] Wang H, Zhang T, Zhu J (2017). A novel mechanism for red emission carbon dots: hydrogen bond dominated molecular states emission. Nanoscale.

[37] Marini A, Muñoz-Losa A, Biancardi A, Mennucci B (2010). What is solvatochromism?. J Phys Chem B.

[38] Sharma A, Gadly T, Gupta A (2016). Origin of excitation dependent fluorescence in carbon nanodots. J Phys Chem Lett.

[39] Reichardt C (1994). Solvatochromic dyes as solvent polarity indicators. Chem Rev.

[40] Chien CT, Li SS, Lai WJ (2012). Tunable photoluminescence from graphene oxide. Angew Chem Int Ed.

[41] Park Y, Yoo J, Lim B (2016). Improving the functionality of carbon nanodots: doping and surface functionalization. J Mater Chem A.

[42] Sharma A, Gadly T, Neogy S (2017). Molecular origin and self-assembly of fluorescent carbon nanodots in polar solvents. J Phys Chem Lett.

[43] Zheng H, Wang Q, Long Y (2011). Enhancing the luminescence of carbon dots with a reduction pathway. Chem Commun.

[44] Bao L, Liu C, Zhang ZL, Pang DW (2015). Photoluminescence-tunable carbon nanodots: surface-state energy-gap tuning. Adv Mater.

[45] Ding H, Yu SB, Wei JS, Xiong HM (2016). Full-color light-emitting carbon dots with a surface-state-controlled luminescence mechanism. ACS Nano.

[46] Zhu S, Zhang J, Tang S (2012). Surface chemistry routes to modulate the photoluminescence of graphene quantum dots: from fluorescence mechanism to up-conversion bioimaging applications. Adv Funct Mater.

[47] Gong X, Liu Y, Yang Z (2017). An “on-off-on” fluorescent nanoprobe for recognition of chromium (VI) and ascorbic acid based on phosphorus/nitrogen dual-doped carbon quantum dot. Anal Chim Acta.

[48] Wang W, Lei W, Xia X (2013). Graphene quantum dots as a fluorescent sensing platform for highly efficient detection of copper (II) ions. Sensors Actuators B Chem.

[49] Dhenadhayalan N, Lin KC (2015). Chemically induced fluorescence switching of carbon-dots and its multiple logic gate implementation. Sci Rep.

[50] Mou M, Wu Y, Niu Q (2017). Aggregation-induced emission properties of hydrothermally synthesized Cu-In-S quantum dots. Chem Commun.

[51] Stockman MI (2008). Spasers explained. Nat Photonics.

