# Supplementary material for: Surface States Induced Photoluminescence Enhancement of Nitrogen-Doped Carbon Dots Via Post-Treatments
Source: Nanoscale Res Lett. 2019 May 24;14:172. doi: 10.1186/s11671-019-3008-9 (PMC6534678; doi:10.1186/s11671-019-3008-9)
Supplement: Supplementary file 1 — Figure S1. (a) XRD pattern and (b) XPS survey of NCDs. Figure S2. Photoluminescence spectra of NCDs in 6 different solvents of (a) water, (b) ethylene glycol (EG), (c) ethanol, (d) dimethyl sulfoxide (DMSO), (e) acetone, and (f) toluene excited by different wavelengths. Figure S3. Relative emission intensity of NCDs. Figure S4. Fluorescent decay curves of NCDs in different solvents. Table S1. Six solvents used in this study. ET (30) polarity parameter of the solvents. Emission peak, absorption band, Stokes shifts, and lifetimes for the CDs dispersed in each kind of solvents. Figure S5. (a) Normalized PL spectra of NCDs treated with different concentrations of NaBH4 ranging from 0 to 0.04 g/mL, λex = 400 nm. (b) PL intensities and (c) peak positions of two peaks deconvoluted from emission spectra of NCDs treated with different concentrations of NaBH4. Figure S6. (a) XPS survey spectrum of NCDs treated by 0.04 g/mL NaBH4. High-resolution (b) C 1s, (c) N 1s, and (d) O 1s spectra. Table S2. XPS results of NCDs in the absence and presence of 0.04 g/mL NaBH4. The measured atomic ratios of C, N, and O were calculated. Figure S7. Fluorescence decay curves of CDs in the absence and presence of 0.04 g/mL NaBH4. Figure S8. Fluorescence decay curves of NCDs in the absence and presence of Ag+ with the concentration of 200 μM (in the HEPES-buffered water solution). Figure S9. TEM image of NCDs treated by Ag+ (200 μM). (DOCX 32773 kb) [file 11671_2019_3008_MOESM1_ESM.docx]

Additional file 1

**Surface states induced photoluminescence enhancement of nitrogen-doped carbon dots via post-treatments**

Xian Wei ^1, 3^, Shiliang Mei ^1, 3^, Dan Yang ^1^, Guilin Zhang ^1^, Fengxian Xie ^1^, Wanlu Zhang^1,*^, Ruiqian Guo ^1, 2,*^

Xian Wei: [16210720118@fudan.edu.cn](mailto:16210720118@fudan.edu.cn). Shiliang Mei: [meishiliang@fudan.edu.cn](mailto:meishiliang@fudan.edu.cn).

Dan Yang: [18210720023@fudan.edu.cn](mailto:18210720023@fudan.edu.cn). Guilin Zhang: [16210720023@fudan.edu.cn](mailto:16210720023@fudan.edu.cn).

Fengxian Xie: [xiefengxian@fudan.edu.cn](mailto:xiefengxian@fudan.edu.cn). Wanlu Zhang: [fdwlzhang@fudan.edu.cn](mailto:fdwlzhang@fudan.edu.cn).

Ruiqian Guo: rqguo@fudan.edu.cn.

1 Engineering Research Center of Advanced Lighting Technology, Ministry of Education; Institute for Electric Light Sources, Fudan University, Shanghai 200433, China

2 Institute of Future Lighting, Academy for Engineering and Technology, Fudan University, Shanghai 200433, China

3 Authors contributed equally.

* Corresponding author.

Tel.: +86 2155664588.

E-mail address: rqguo@fudan.edu.cn, fd[wlzhang@fudan.edu.cn](mailto:wlzhang@fudan.edu.cn)


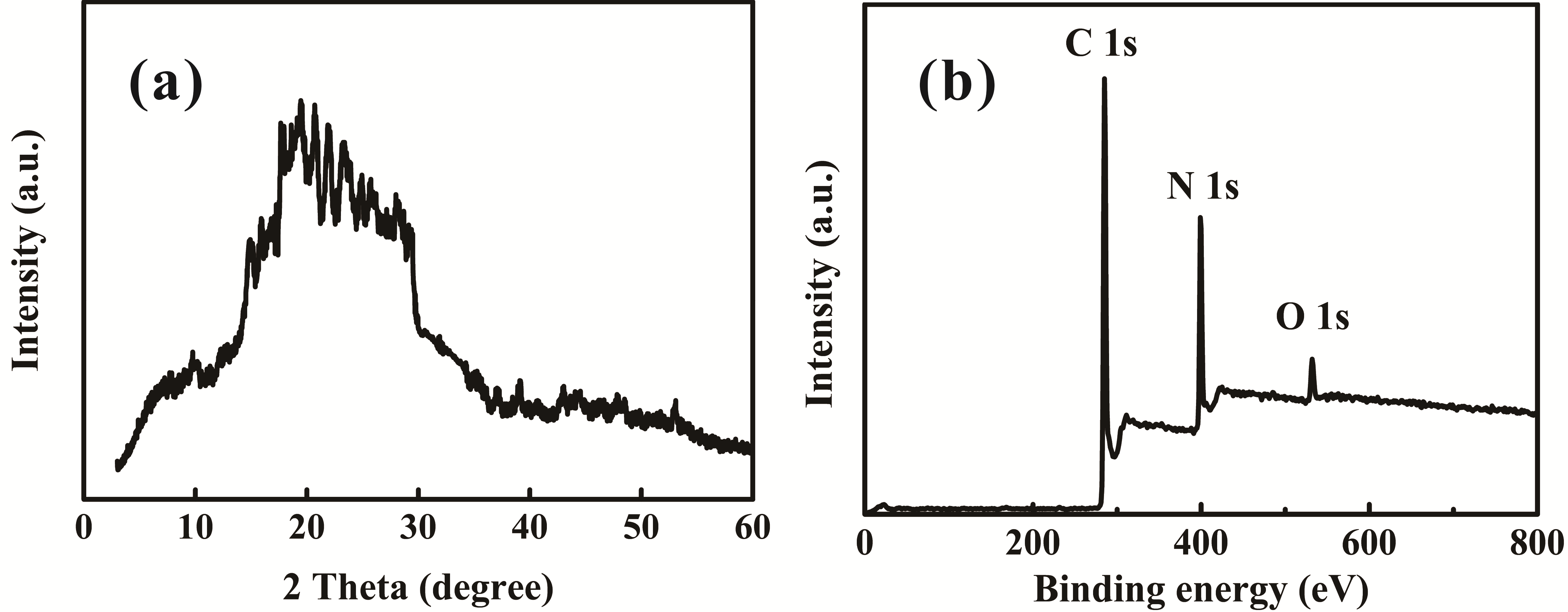


Figure S1 (a) XRD pattern and (b) XPS survey of NCDs.


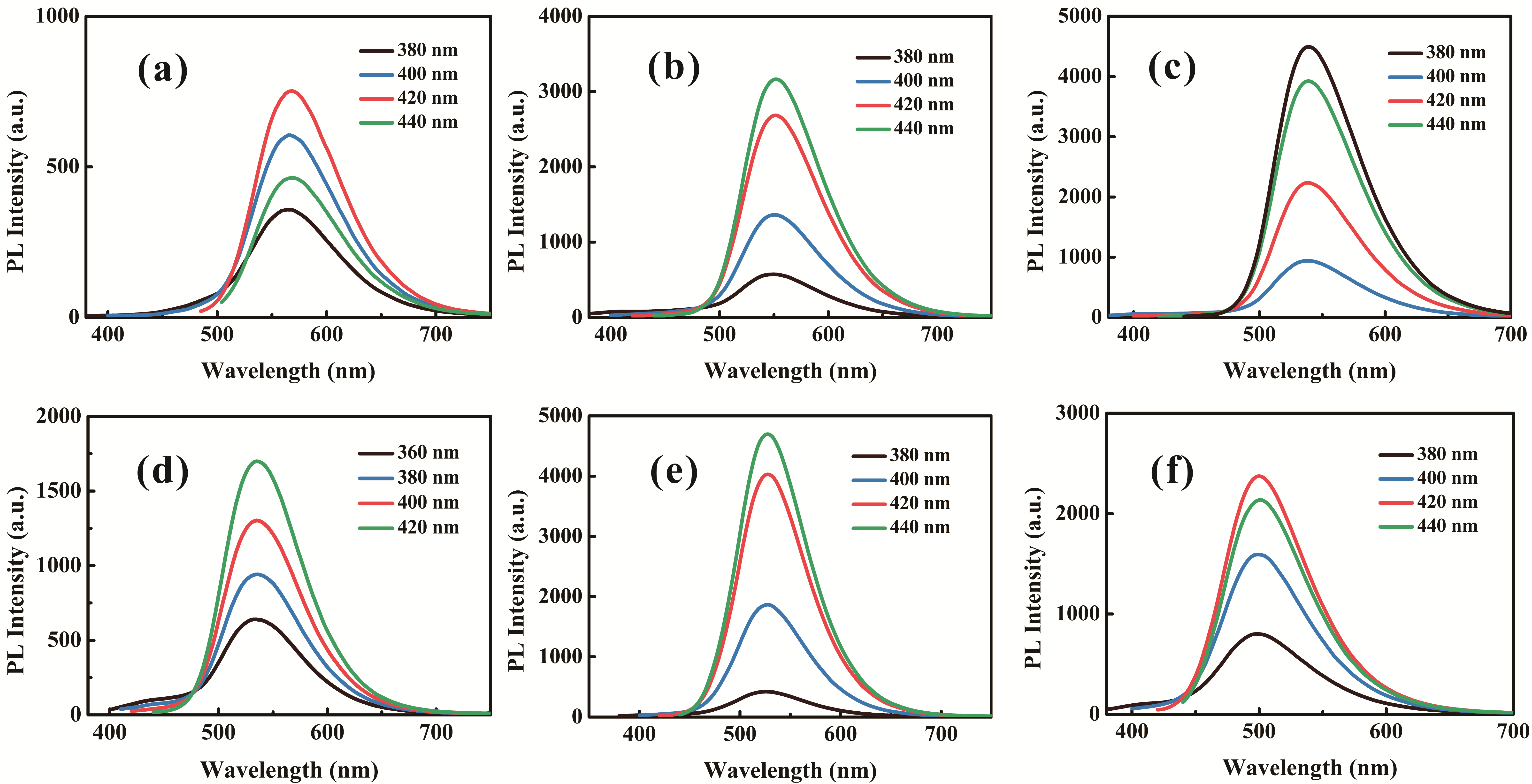


Figure S2 Photoluminescence spectra of NCDs in 6 different solvents of (a) water, (b) ethylene glycol (EG), (c) ethanol, (d) dimethyl sulfoxide (DMSO), (e) acetone and (f) toluene excited by different wavelengths.


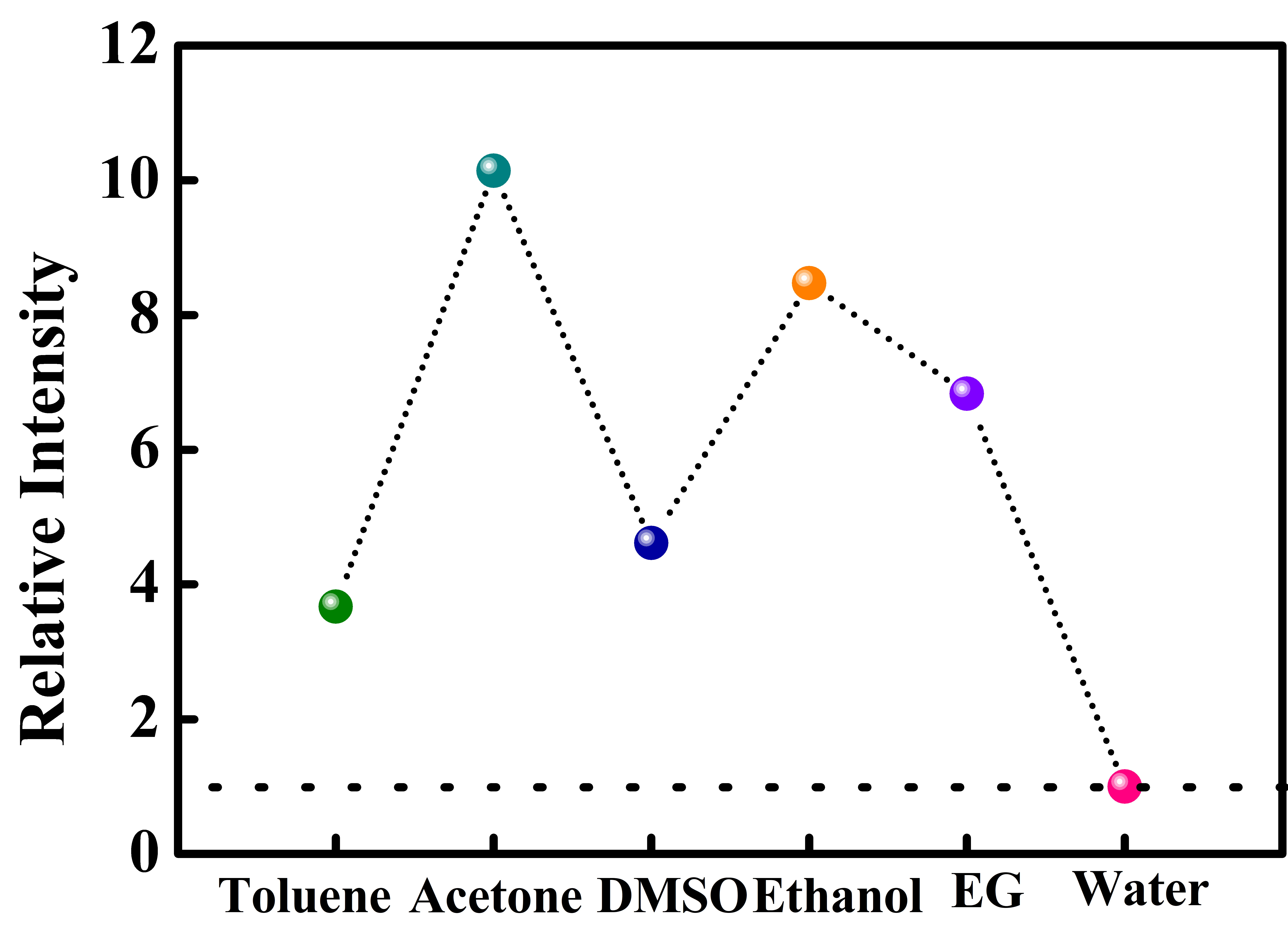


Figure S3 Relative emission intensity of NCDs.


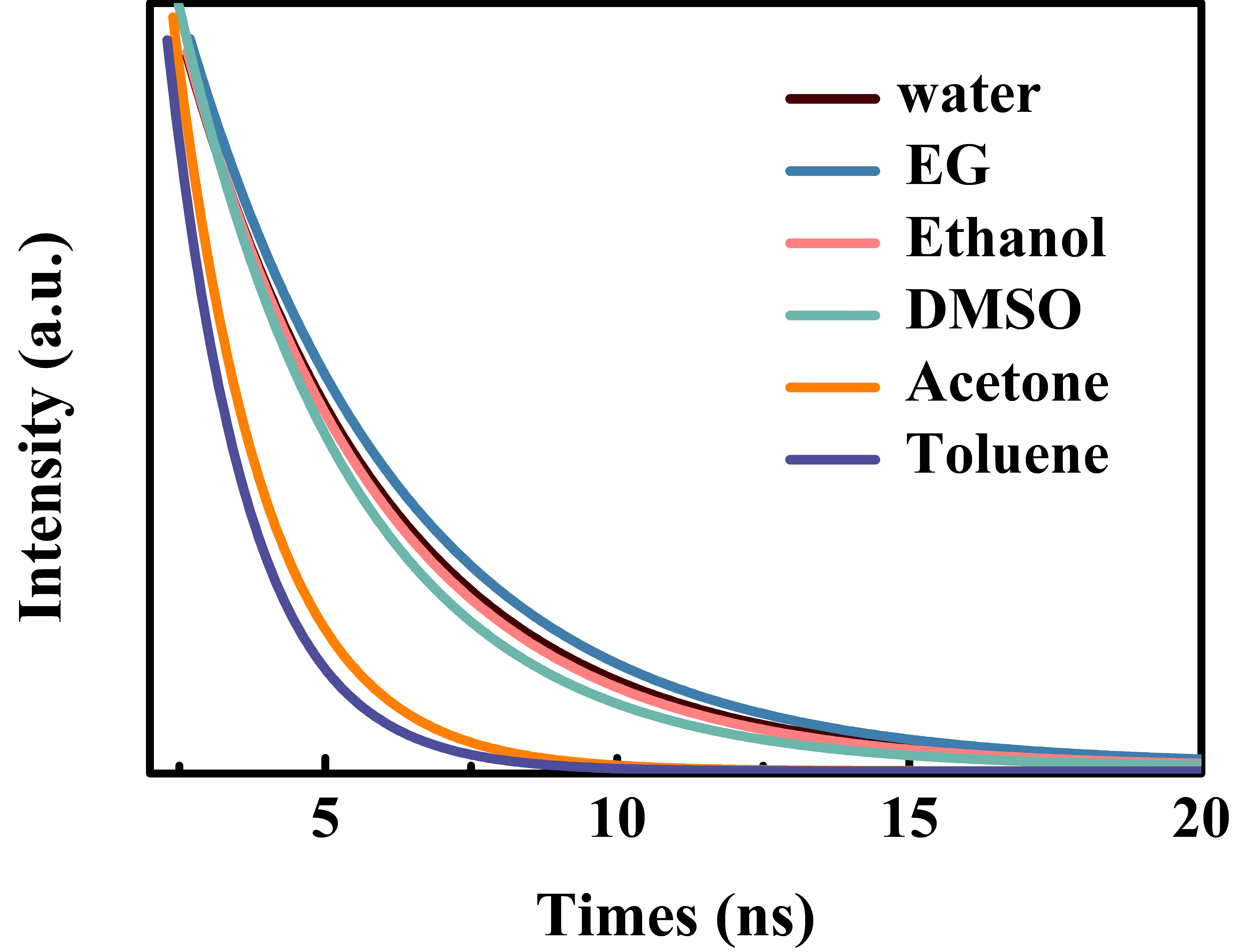


Figure S4 Fluorescent decay curves of NCDs in different solvents.

Table S1 Six solvents used in this study. E_T_ (30) polarity parameter of the solvents. Emission peak, absorption band, Stokes shifts and lifetimes for the CDs dispersed in each kinds of solvents.


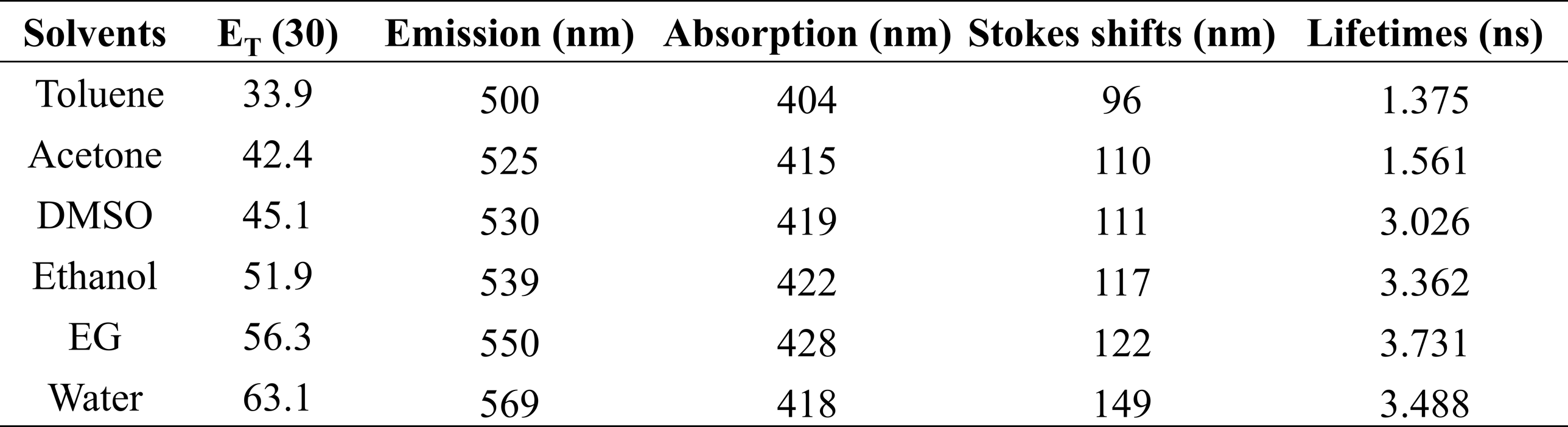

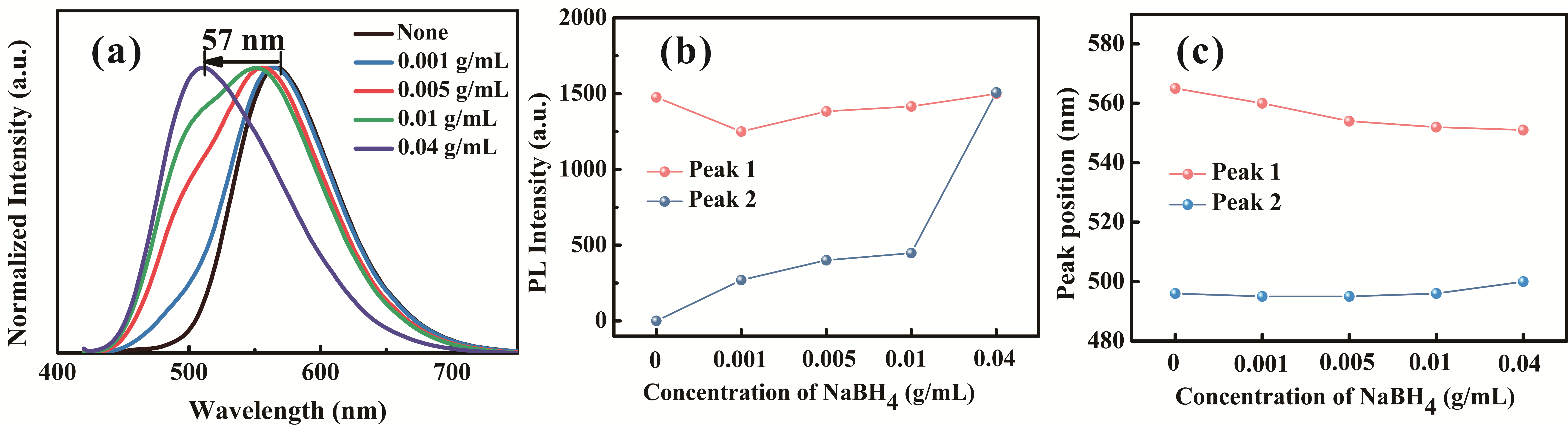


Figure S5 (a) Normalized PL spectra of NCDs treated with different concentration of NaBH_4_ ranging from 0 to 0.04 g/mL, λ_ex_=400 nm. (b) PL intensities and (c) peak positions of two peaks deconvoluted from emission spectra of NCDs treated with different concentration of NaBH_4_.


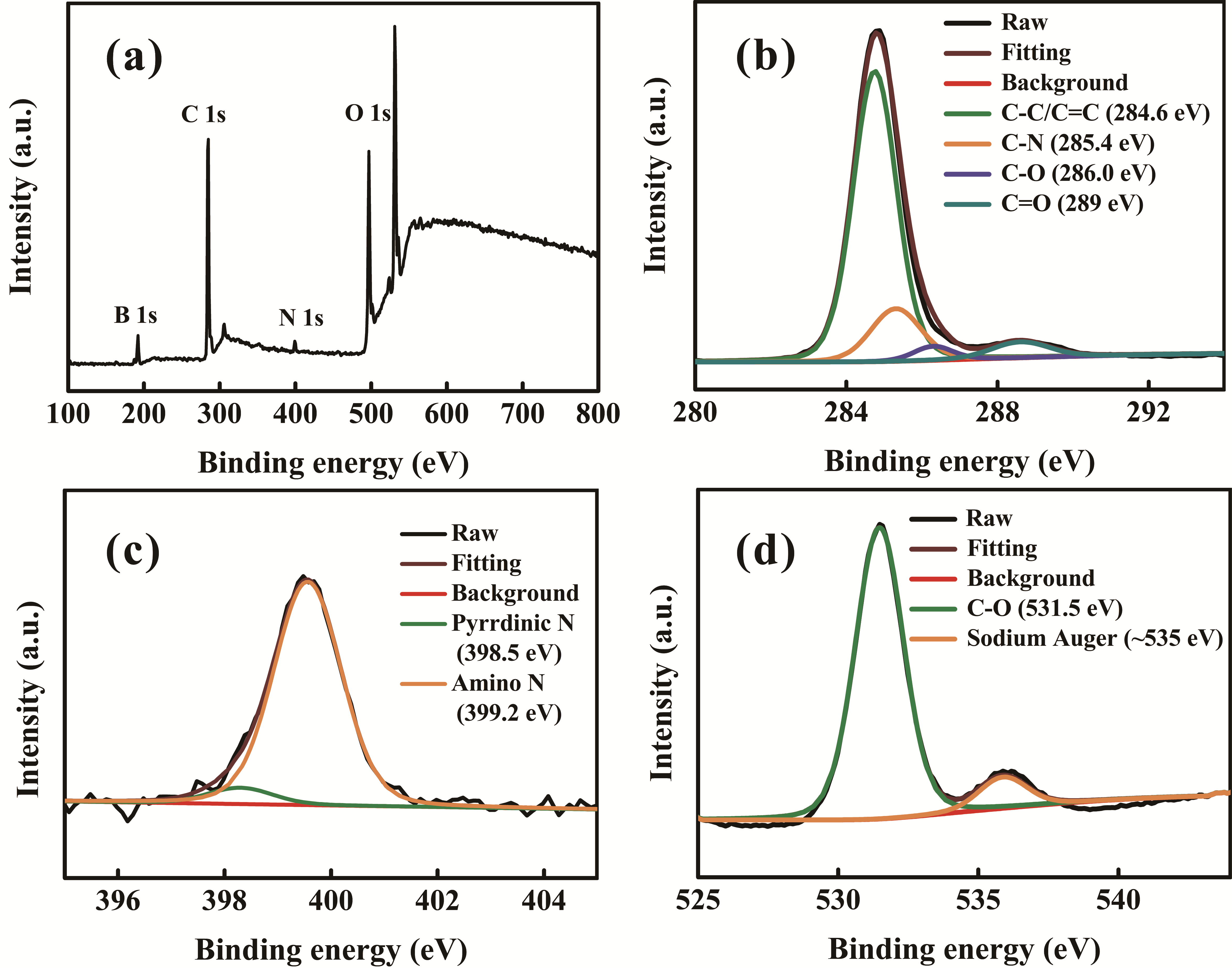


Figure S6 (a) XPS survey spectrum of NCDs treated by 0.04 g/mL NaBH_4_. High-resolution (b) C 1s, (c) N 1s and (d) O 1s spectra.

Table S2 XPS results of NCDs in the absence and presence of 0.04 g/mL NaBH_4_. The measured atomic ratios of C, N and O were calculated.


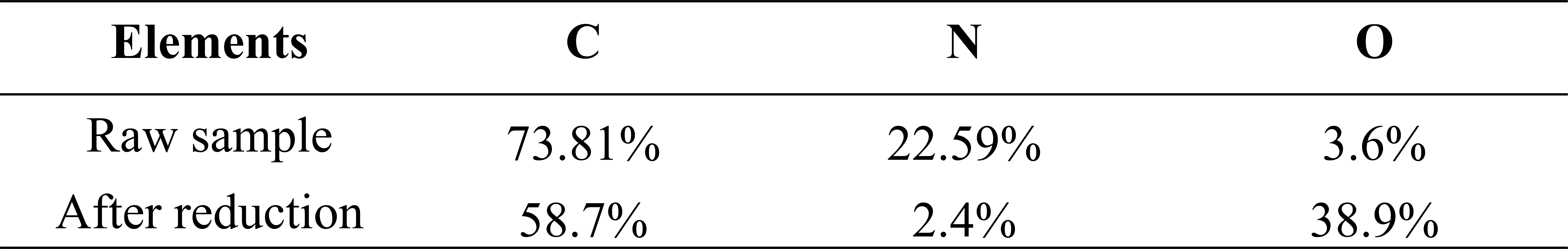

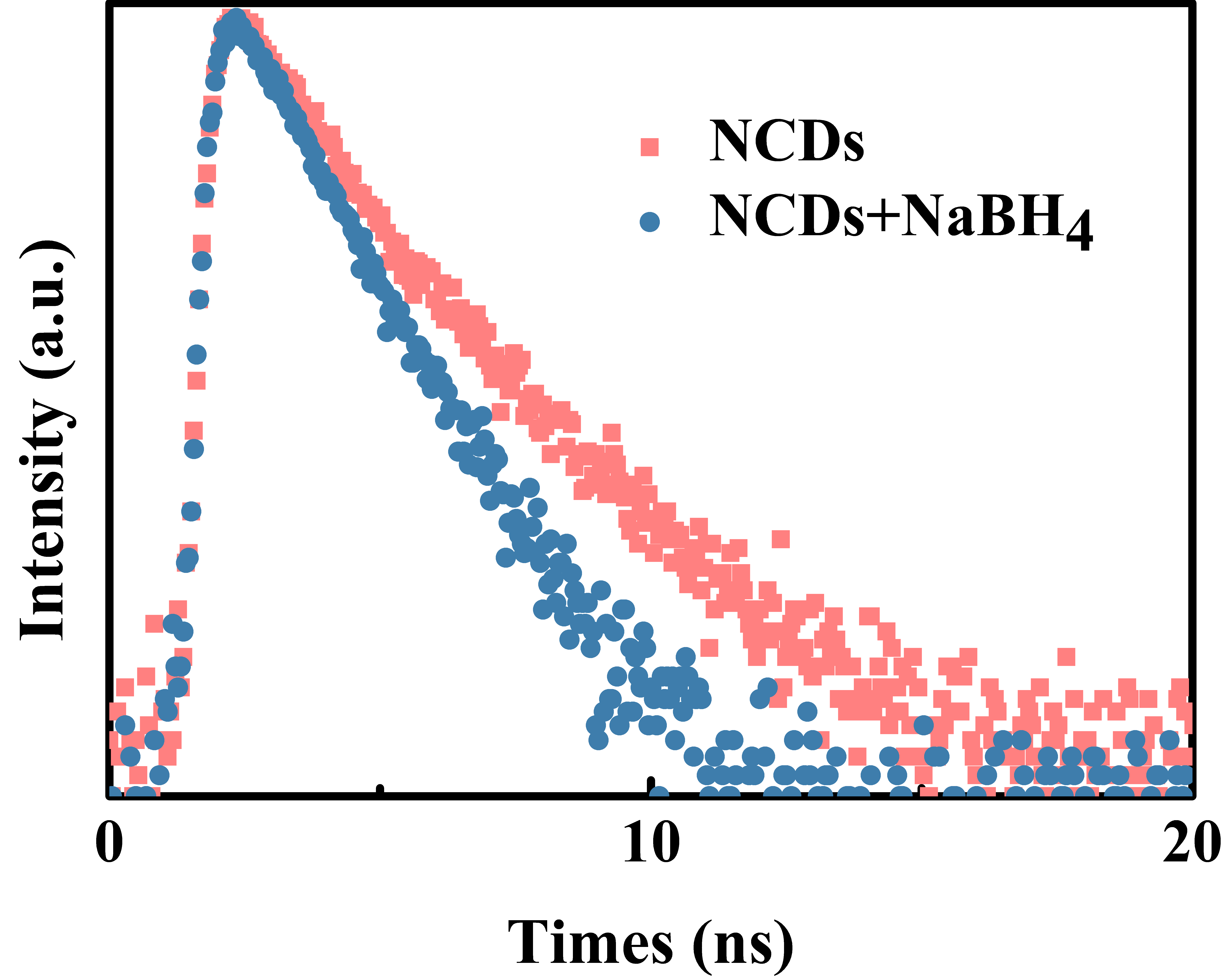


Figure S7 Fluorescence decay curves of CDs in the absence and presence of 0.04 g/mL NaBH_4_.


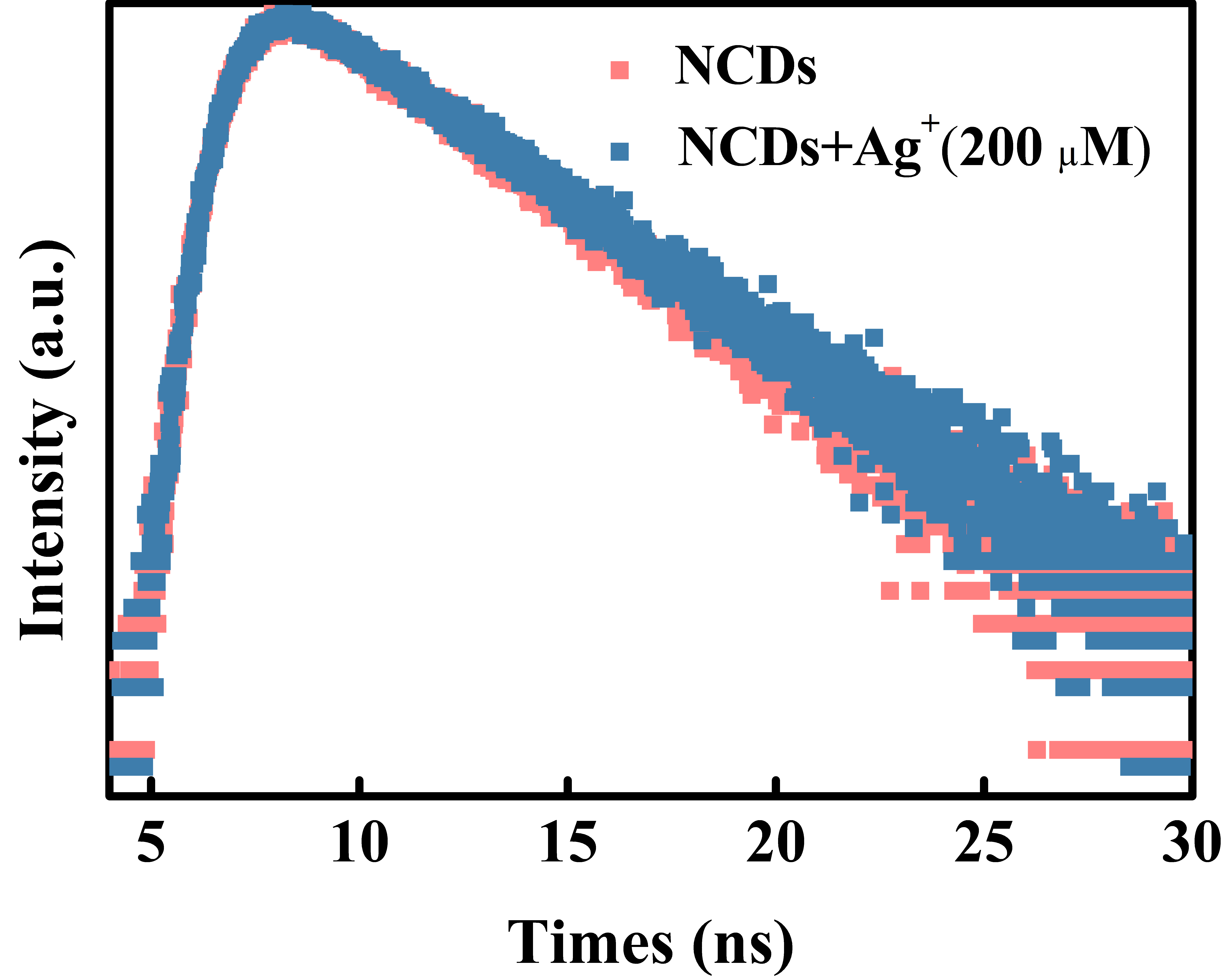


Figure S8 Fluorescence decay curves of NCDs in the absence and presence of Ag^+^ with the concentration of 200 μM. (in the HEPES-buﬀered water solution).

Figure S9 TEM image of NCDs treated by Ag^+^ (200 μM)
